# Leveraging Genetic Findings for Precision Medicine in Vasculitis

**DOI:** 10.3389/fimmu.2019.01796

**Published:** 2019-08-02

**Authors:** Marialbert Acosta-Herrera, Miguel A. González-Gay, Javier Martín, Ana Márquez

**Affiliations:** ^1^Instituto de Parasitología y Biomedicina “López-Neyra,” CSIC, Granada, Spain; ^2^Division of Rheumatology and Epidemiology, Genetics and Atherosclerosis Research Group on Systemic Inflammatory Diseases, Hospital Universitario Marqués de Valdecilla, IDIVAL, University of Cantabria, Santander, Spain; ^3^Systemic Autoimmune Disease Unit, Hospital Clínico San Cecilio, Instituto de Investigación Biosanitaria ibs.GRANADA, Granada, Spain

**Keywords:** systemic vasculitis, polymorphism, genome-wide association studies, immunochip, precision medicine

## Abstract

Vasculitides are a heterogeneous group of low frequent disorders, mainly characterized by the inflammation of blood vessels that narrows or occlude the lumen and limits the blood flow, leading eventually to significant tissue and organ damage. These disorders are classified depending on the size of the affected blood vessels in large, medium, and small vessel vasculitis. Currently, it is known that these syndromes show a complex etiology in which both environmental and genetic factors play a major role in their development. So far, these conditions are not curable and the therapeutic approaches are mainly symptomatic. Moreover, a percentage of the patients do not adequately respond to standard treatments. Over the last years, numerous genetic studies have been carried out to identify susceptibility *loci* and biological pathways involved in vasculitis pathogenesis as well as potential genetic predictors of treatment response. The ultimate goal of these studies is to identify new therapeutic targets and to improve the use of existing drugs to achieve more effective treatments. This review will focus on the main advances made in the field of genetics and pharmacogenetics of vasculitis and their potential application for ameliorating long-term outcomes in patient management and in the development of precision medicine.

## Introduction

Systemic vasculitides represent a heterogeneous group of chronic diseases characterized by the inflammation of the blood vessels. These disorders are classified according to the diameter of the affected vessels in large, medium and small vessel vasculitis, and may affect one or several organs and tissues of the body, resulting in different clinical presentations. In the past years, considerable therapeutic advances have been made in the treatment of vasculitis; however, the lack of appropriate therapeutic response and the appearance of side effects remain a major concern (1).

**Figure 1:**
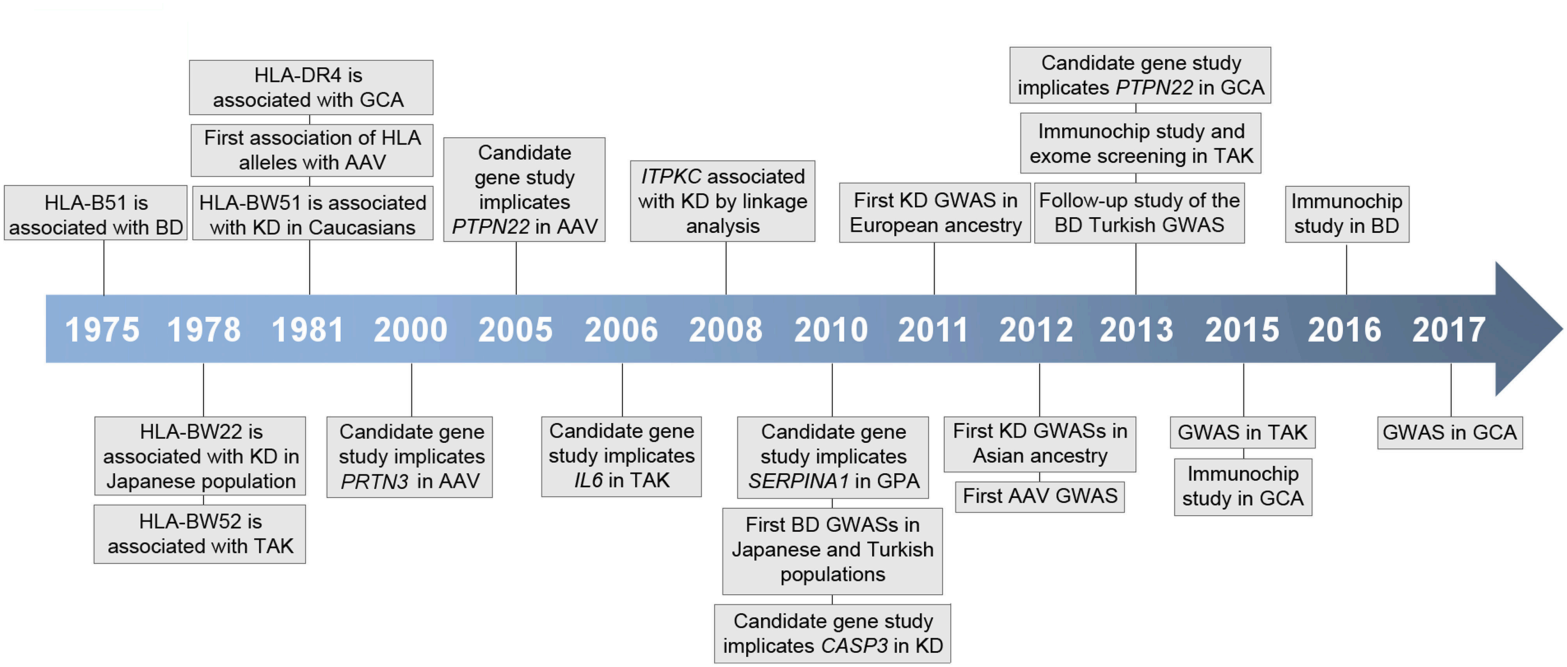
Timeline representing key events in vasculitis genetic research. HLA, human histocompatibility complex; BD, Behçet's disease; GCA, giant cell arteritis; AAV, ANCA-associated vasculitis; KD, Kawasaki's disease; TAK, Takayasu arteritis; GPA, granulomatosis with polyangiitis; GWAS, genome-wide association study.

Although the specific mechanisms underlying vasculitis are not fully understood, it is currently known that these conditions show a complex etiology in which both genetic and environmental factors appear to contribute to their pathogenesis (2). In recent years, our knowledge of the genetic landscape of vasculitis has experienced a significant increase, mainly due to the development of large-scale genetic scans, including genome-wide association studies (GWASs) and Immunochip studies, focused on analyzing single-nucleotide polymorphisms (SNPs) in cases and controls (Figure 1). In addition to the human leukocyte antigen (HLA) region, which represents the strongest association in vasculitis, multiple *loci* located outside the HLA have been shown to play a role in the genetic predisposition to these disorders (Table 1).

**Table 1:** Non-HLA loci associated with vasculitis at genome-wide significance level.

**Type of vasculitis**	**Susceptibility locus**	**Chromosomic region**	**Population**	**Approach**	**References**
TAK	*FCGR2A*	1q23.3	Turkish, European-American	Immunochip	(3)
	*IL12B*	5q33.3	Turkish, European-American, Japanese	GWAS	(3, 4)
	*IL6*	7p15.3	Turkish, European-American	GWAS	(5)
	*LILR3B*	19q13.42	Turkish, European-American	GWAS	(5)
	*PSMG1*	21q22.2	Turkish, European-American	GWAS	(5)
GCA	*PTPN22*	1p13.2	European	Candidate-gene, Immunochip	(6, 7)
	*PLG*	6q26	European	GWAS	(8)
	*P4HA2*	5q31.1	European	GWAS	(8)
AAV	*SERPINA1*	14q32.13	European	Candidate-gene, GWAS	(9–11)
	*PRTN3*	19p13.3	European	Candidate-gene, GWAS	(9, 10, 12)
	*PTPN22*	1p13.2	European	Candidate-gene, GWAS	(10, 13–15)
	*SEMA6A*	5q23.1	European	GWAS	(13)
BD	*IL10*	1q32.1	Japanese, Turkish	GWAS	(16, 17)
	*IL23R/IL12RB2*	1p31.3	Japanese, Turkish, European	GWAS, Immunochip	(16–18)
	*CCR1/CCR3*	3p21.31	Turkish	GWAS follow-up	(19)
	*STAT4*	2q32.2-q32.3	Turkish	GWAS follow-up	(19)
	*ERAP1*	5q15	Turkish	GWAS follow-up	(19)
	*KLRC4*	12p13.2	Turkish	GWAS follow-up	(19)
	*GIMAP4*	7q36.1	Korean	GWAS	(20)
	*IL12A*	3q25.33	European, Middle East and Turkish	GWAS, Immunochip	(18, 21)
	*JRKL/CNTN5*	11q22.1	European	Immunochip	(18)
KD	*ITPKC*	19q13.2	European, Asian	GWAS	(22)
	*FCGR2A*	1q23.3	European, Asian	GWAS	(22)
	*CASP3*	4q35.1	European, Japanese	Candidate-gene	(23)
	*BLK*	8p23.1	Han Chinese, Japanese, Korean	GWAS	(24–26)
	*CD40*	20q13.12	Han Chinese, Japanese	GWAS	(25, 26)

*TAK, Takayasu arteritis; GCA, giant cell arteritis; AAV, Anti-neutrophil cytoplasmic antibody-associated vasculitis; BD, Behçet's disease; KD, Kawasaki's disease; GWAS, genome-wide association study*.

Identification of genes and molecular pathways deregulated in vasculitis is crucial to better understand disease pathogenesis and for the development of more effective therapeutic approaches. In this sense, Nelson et al. (27) demonstrated that a drug with genetic support has twice the possibilities of going from phase I to approval in the different phases of drug development, than those drugs without genetic support. The authors found that genes associated with a broad spectrum of human diseases were significantly enriched in target genes for drugs approved in the United States or the European Union, highlighting the importance of the provided genetic knowledge in different drug mechanisms. In addition, genetic studies not only have the potential to identify molecular targets for new therapies, but they also allow us to determine the best way to administer current treatments. In this regard, several pharmacogenetic studies based on candidate genes have identified a number of genetic variants influencing treatment response in different vasculitides (Table 2).

**Table 2:** Genes associated with treatment response in vasculitis.

**Vasculitis**	**Locus**	**Region**	**Treatment**	**Population**	**Approach**	**References**
KD	*ITPKC*	19q13.2	IVIG	Japanese, Taiwanese	Candidate-gene	(28, 29)
	*CASP3*	4q35.1	IVIG	Japanese, Taiwanese	Candidate-gene	(28, 29)
	*FCGR2C*	1q23.3	IVIG	European, Asian, African-American, Hispanic	Candidate-gene	(30)
	*FCGR3B*	1q23.3	IVIG	European, Asian, African-American, Hispanic	Candidate-gene	(30, 31)
	*FCGR2B*	1q23.3	IVIG	European, Asian, African-American, Hispanic	Candidate-gene	(32)
	*CCL17*	16q21	IVIG	Taiwanese	Candidate-gene	(33)
	*CCR5*	3p21.31	IVIG	Japanese	Candidate-gene	(34)
	*CCL3L1*	17q21.1	IVIG	Japanese	Candidate-gene	(34)
	*IL1B*	2q14.1	IVIG	Taiwanese	Candidate-gene	(35)
	*IFNG*	12q15	IVIG	Taiwanese	Candidate-gene	(36)
	*HMGB1*	13q12.3	IVIG	Korean	Candidate-gene	(37)
	*BCL2L11*	2q13	IVIG	Korean	GWAS	(38)
	*STX1B*	16p11.2	IVIG	European	Immunochip	(39)
	*BAZ1A/C14orf19*	14q13.1-q13.2	IVIG	European	Immunochip	(39)
	*SAMD9L*	7q21.2	IVIG	Korean	GWAS	(40)
AAV	HLA-DRB1^*^0405	6p21.32	Remission induction therapy	China	Candidate-gene	(41)
	*FCGR2A*	1q23.3	Rituximab or cyclophosphamide	–	Candidate-gene	(42)
	*TNFSF13B*	13q33.3	Rituximab	European	Candidate-gene	(43)
BD	*ABCB1*	7q21.12	Colchicine	Turkish	Candidate-gene	(44)
	*MTHFR*	1p36.22	Colchicine	Turkish	Candidate-gene	(45)

*KD, Kawasaki's disease; AAV, Anti-neutrophil cytoplasmic antibody-associated vasculitis; BD, Behçet's disease; IVIG, intravenous immunoglobulin; GWAS, genome-wide association study*.

This review aims to provide an update of the main findings obtained from genetic and pharmacogenetic studies as well as their potential application to precision medicine in vasculitis.

## Contribution of Genetics to New Therapeutic Approaches in Vasculitis

### Takayasu Arteritis

Takayasu arteritis (TAK) is a chronic vasculitis characterized by granulomatous inflammation of large vessels, predominantly the aortic arch and its branches, which results in non-specific constitutional symptoms, such as fever and weight loss, and serious complications, including arterial stenosis, occlusion and aneurysm. This disease affects mainly young females with a higher incidence in Asia and Latin America (46).

Genetic studies have shown that the HLA region represents the main genetic risk factor in TAK. Specifically, an association at the genome-wide significance level between the classical allele HLA-B^*^52:01 and this vasculitis has been reported in TAK patients from Japanese, Turkish and European-American origin (3, 47), and confirmed in Greek, Mexican Mestizo, India, Thai, and Korean populations (48–52). Moreover, independent associations within the HLA class II region, specifically with the DRB1^*^07 classical allele and DRB1/DQB1 polymorphisms, have also been reported (3, 53, 54); however, additional studies in well-powered populations are required to confirm these findings.

Outside the HLA region, five *loci* have been consistently associated with TAK through three large-scale genetic analyses (3–5), *IL6* (interleukin 6), encoding a cytokine that plays a crucial role in the immune response by regulating the balance between Th17 cells and regulatory T cells (Treg) (55); *LILRB3* (leukocyte immunoglobulin like receptor B3), which encodes a protein that binds to HLA class I molecules to inhibit immune cell stimulation (56); *IL12B*, encoding the p40 subunit of IL-12 and IL-23, two cytokines with a key role in the inflammatory responses mediated by Th1 and Th17 cells, respectively (57); *FCGR2A* (Fc fragment of IgG receptor IIa) that encodes an immunoglobulin Fcg receptor (FcgR), which has a relevant role in humoral immunity by participating in modulation of antibody production by B cells, phagocytosis, and clearing of immune complexes (58); and an intergenic locus on chromosome 21q22 near *PSMG1* (proteasome assembly chaperone 1).

Interestingly, some genes associated with TAK are being explored as therapeutic targets for this vasculitis. On one hand, tocilizumab, a humanized monoclonal antibody against IL-6 receptor (IL-6R), has shown clinical efficacy in TAK patients in several case series studies (59). This efficiency was confirmed in a prospective clinical trial evaluating tocilizumab in refractory TAK, although the primary end-point was not met, probably due to the low number of individuals included in this study (60). A phase III clinical trial evaluating this biological agent as a first-line therapy in TAK patients is currently underway (NCT02101333).

On the other hand, administration of ustekinumab, a monoclonal antibody to the p40 subunit common to IL-12 and IL-23, to patients with active TAK achieved decrease of inflammatory markers but did not improve vascular lesions in a pilot clinical trial (61). In a more recent study, this drug was used to treat a patient with refractory TAK and psoriasis (for which this drug is approved), two diseases that share the genetic risk locus *IL12B*, with satisfactory results (62). Ustekinumab allowed a significant reduction in glucocorticoid dose and full reduction of vessel wall thickness, thus demonstrating the usefulness of drug repositioning based on the existence of a common genetic component.

Moreover, several evidences, including the association observed between *FCGR2A* and TAK, indicate that, in addition to T lymphocytes, B cells are also involved in the pathogenesis of this vasculitis. In this regard, depletion of B cells using rituximab, a chimeric anti-CD20 monoclonal antibody, has been shown to be effective in a case series study, achieving clinical and laboratory remission (63). Nevertheless, a randomized control trial is needed in order to confirm the efficacy of rituximab in patients with TAK.

### Giant Cell Arteritis

Giant cell arteritis (GCA) is a vasculitis characterized by chronic inflammation of medium- and large-sized blood vessels, mainly the aorta and external carotid arteries and their branches. A severe complication of this disorder is the occlusion of the ophthalmic artery, which leads to acute and irreversible blindness. GCA represents the most frequent vasculitis in elderly individuals from Western countries affecting predominantly women and people over 50 years of age (64).

In the last years, a high number of candidate gene association studies have been performed in GCA, most of them focused on analyzing genes encoding inflammatory cytokines (65). These studies identified the HLA class II region, specifically the classical allele DRB1^*^04, as the main genetic risk factor in GCA. However, both the low sample size and the lack of replication cohorts of these studies have been limiting factors in the identification of robust genetic associations outside the HLA region. Nevertheless, some of the non-HLA loci associated with this vasculitis using candidate-gene approaches were replicated in different populations (66–71) and, therefore, they represent potential genetic risk factors in GCA, including *IL33*, which encodes a member of the IL-1 family involved in pro-inflammatory cytokines production, angiogenesis and vascular permeability (72, 73); *IL17A*, encoding a pro-inflammatory cytokine with a relevant role in the differentiation of Th17 lymphocytes (74); *VEGF* (vascular endothelial growth factor), encoding a proangiogenic mediator (75); and *NLRP1* (NLR family pyrin domain containing 1), encoding a protein implicated in the formation of the inflammasome, which activates caspases 1 and 5 leading to the activation of pro-inflammatory cytokines such as IL-1β and IL-18 (76).

More recently, the emergence of massive genotyping platforms and the formation of a large consortium focused on the study of the genetic basis of GCA have allowed a significant progress in the identification of this genetic component. Until now, two large-scale genetic studies, a GWAS and an Immunochip, have been performed in GCA (6, 8). Both of them have confirmed the classical allele HLA-DRB1^*^04 as the most consistent association with this vasculitis. In addition, several non-HLA loci have been also found to play a role in the GCA genetic predisposition, including *PTPN22* (protein tyrosine phosphatase non-receptor type 22), *PLG* (plasminogen), and *P4HA2* (prolyl 4-hydroxylase subunit alpha 2).

The association between *PTPN22* and GCA was initially identified in a candidate-gene association study (7) and subsequently confirmed by using the Immunochip strategy (6). This gene encodes LYP, a tyrosine phosphatase involved in several immune signaling pathways, such as the T cell receptor (TCR) pathway and the humoral activity of B cells. The strongest signal within this *locus* corresponds to a functional variant (rs2476601), previously associated with multiple immune-mediated disorders, that results in a non-synonymous arginine to tryptophan amino acid change (R620W). It has been described that carrying the rs2476601 risk allele results in enhanced B lymphocyte autoreactivity, deregulated TCR signaling, and reduced capacity for TLR-induced type 1 interferon (IFN) production (77). On the other hand, *PLG*, encoding plasminogen, is involved in different processes relevant for GCA, such as angiogenesis, lymphocyte recruitment, and production of inflammatory mediators, including tumor necrosis factor alpha (TNF-α) and IL-6 (78), and *P4HA2*, encoding an isoform of the alpha subunit of the collagen prolyl 4-hydroxylase, is an important hypoxia response gene whose expression is induced by hypoxia-inducible factor-1 (HIF-1), which also induces the expression of other genes involved in GCA such as *IL6, MMP9* (matrix metallopeptidase 9), and *VEGF* (79).

These genetic findings, together with other lines of evidence, have contributed to the identification of several molecular pathways implicated in the GCA pathogenesis. Currently, it is known that both Th1 and Th17 cells are relevant player in GCA with two main cytokine clusters contributing to the local inflammation, the IL-6/IL-17 and the IL-12/IFN-γ axes (74). Interestingly, whereas the inflammatory activity of the IL-6/IL-17 cytokine cluster seems to be affected by glucocorticoid treatment, the IL-12/IFN-γ cytokine cluster is resistant to this therapy. This, together with the adverse events associated with long-term glucocorticoids use, has led to the search for new therapeutical agents.

Considering the major role of IL-6 in the pathogenesis of GCA, the potential use of tocilizumab in the treatment of this vasculitis has been explored. IL-6 inhibition has shown clinical efficacy in several randomized controlled trials (80, 81), thus representing a promising therapeutic strategy for this type of vasculitis. Indeed, tocilizumab has been recently approved to treat GCA by the United States Food and Drug Administration (FDA). Furthermore, other biological agents, such as ustekinumab and abatacept, a fusion protein comprising the Fc region of IgG1 and the extracellular domain of cytotoxic T lymphocyte antigen 4 (CTLA4) that inhibits the co-stimulatory signal required for T cell activation, have also shown encouraging but more moderate results (82, 83). Better powered studies are required in order to evaluate the efficacy of these drugs in GCA.

Finally, the potential therapeutic application of two monoclonal antibodies, anakinra and secukinumab, targeted against IL-1β receptor and IL-17A (one of the genes associated with GCA), respectively, is currently under investigation (NCT02902731 and NCT03765788). Both cytokines are crucial for the differentiation of Th17 cells and, therefore, their inhibition could be a therapeutic option in patients with GCA.

### ANCA-Associated Vasculitis

Anti-neutrophil cytoplasmic antibody (ANCA)-associated vasculitis (AAV) is a group of disorders characterized by necrosing inflammation of small vessels, including arterioles, capillaries and venules, that comprises three separate conditions, granulomatosis with polyangiitis (GPA), microscopic polyangiitis (MPA), and eosinophilic granulomatosis with polyangiitis (EGPA). AAV frequently affects small vessels in the respiratory tract and kidneys and is characterized by the presence of antibodies directed against two proteins, proteinase 3 (PR3) and myeloperoxidase (MPO), located on the membrane of monocytes and neutrophils (84).

Both candidate gene association studies and GWASs published in last years have identified several loci associated with these forms of vasculitis (2). Specifically, three GWASs on AAV have been performed so far (9, 10, 13), one in European patients with GPA and MPA and two in North American patients of European descent (one including patients with GPA and the other one including patients with GPA and MPA). Interestingly, these studies have shown that the genetic background of AAV depends on auto-antibody specificity rather than clinically defined disorders. In this regard, different HLA genes have been associated with the different ANCA subgroups; whereas polymorphisms within the *DPB1* and *DPA1* genes appeared to be associated with PR3-ANCA-positive patients, *DQB1* showed a specific effect in the MPO-ANCA subgroup (10).

Additionally, four non-HLA genetic *loci, SERPINA1* (serpin family A member 1), *PRTN3* (proteinase 3), *PTPN22*, and *SEMA6A* (semaphorin 6A) have been associated with AAV at genome-wide significance level (9, 10, 13), the first two showing a specific association with the subgroup of patients positive for PR3-ANCA.

*SERPINA1* encodes α1-antitrypsin (α1AT), an inhibitor of the serine proteases, including proteinase 3. The association between this gene and AAV was initially described in a candidate gene study (11), in which a functional genetic variant known to cause a deficient production of α1-AT appeared to be associated with GPA, and subsequently confirmed by GWAS (9, 10). It has been proposed that, since PR3 is a target of α1AT, a decreased production of this inhibitor could result in higher levels of circulating PR3, leading to the synthesis of anti-PR3 ANCA (11).

Regarding *PRTN3*, the role of this gene in AAV was described in a candidate gene association study, in which a genetic variant affecting a putative transcription factor-binding site was associated with GPA (12). Subsequently, two of the GWASs performed in AAV have confirmed this association (9, 10). Interestingly, the most recent GWAS reported that the lead SNP at this gene (rs62132293), which is in almost complete linkage disequilibrium (LD) with that described in the original study, acts as an expression-quantitative trait locus (eQTL) that results in an increased expression of *PRTN3* in neutrophils (10).

The protein encoded by *SEMA6A* has been characterized as a critical regulator of angiogenesis by modulating VEGF signaling (85). Nevertheless, it should be considered that, although polymorphisms within this locus showed genome-wide significance (13), this association was not subsequently validated in a replication study performed in a well-powered cohort of European AAV patients (including GPA, MPA, and EGPA cases) (86) or in the subsequent GWAS carried out by the same group (10). Therefore, further genetic association studies are needed to confirm the role of *SEMA6A* in AAV.

Finally, *PTPN22* has been consistently associated with both GPA and MPA by candidate gene and genome-wide studies (10, 13–15). The highest signal within the *PTPN22* locus lies on the R620W functional variant, the same one associated with GCA, thus pointing to a pleiotropic effect of this polymorphism in both vasculitis.

On the other hand, polymorphisms within the *CTLA4* locus, encoding a protein which transmits an inhibitory signal to T cells by blocking the interaction between CD28 on the T cell and CD80 or CD86 on the antigen-presenting cell, have been implicated in AAV by candidate gene analyses in different populations (87–92) and have shown suggestive association in GWASs (9, 13), thus supporting the idea that this locus represents a genetic risk factor for AAV.

Regarding EGPA, no GWAS has been published in this disease so far and the few associations reported to date have been identified using a candidate-gene strategy. In this regard, an early candidate-gene study reported an association between EGPA and the HLA-DRB4 gene (93), which highlights the existence of different HLA associations for the different AAV subgroups. In addition, polymorphisms within several non-HLA genes, including *FCGR3B* and *IL10*, have also been associated with this vasculitis (94, 95). On the other hand, a role of eotaxin-3 in the EGPA pathogenesis was proposed. Interestingly, serum levels of this molecule were found to be increased in active patients and correlated with eosinophil counts, total immunoglobulin E (IgE) levels and acute-phase parameters (96, 97). However, a genetic study failed to identify an association between the gene encoding this molecule, *CCL26*, and EGPA, maybe due to a lack of statistical power (97). Therefore, the role of this gene as a susceptibility factor for EGPA remains to be clarified.

Although we are still far from fully understanding the pathogenic mechanisms implicated in AAV, genetic studies are contributing to their elucidation. Insights into these pathogenic pathways have opened new strategies for biological treatment. In this line, the central role that ANCA-mediated neutrophil activation plays in these disorders has led to the therapeutic use of B cell depleting drugs for AAV. Rituximab has proved to be highly effective for both remission induction and maintenance treatment (98), representing one of the major breakthroughs of the last decade in the treatment of these vasculitides. Additionally, the therapeutic potential of other B cell-targeting agents, such as belimumab, is being evaluated (NCT01663623). Belimumab is a humanized monoclonal antibody against BAFF, a potent B cell activator, which represents an interesting target for AAV treatment, since it has been reported to increase the production of PR3-ANCA in GPA patients (99).

On the other hand, B cells require T cell help to differentiate into antigen-specific Ig-producing plasma cells. Therefore, blockade of the co-stimulation signal required for full T cell activation using abatacept (CTLA4-Ig) is also an interesting treatment option that has shown clinical efficacy in an open-label clinical trial (100).

### Behçet's Disease

Behçet's disease (BD) is an inflammatory disorder that may affect arteries and veins of all sizes. It is characterized by heterogeneous clinical manifestations, including oral and genital ulcers, which are the hallmark lesions of this vasculitis, as well as vascular, gastrointestinal, articular, and central nervous system manifestations. This condition shows a male preponderance and is more frequent in the Middle East and Asia (101).

BD is the vasculitis that has benefited most from the genome-wide era, with five large-scale genetic studies performed on this disorder so far, four GWASs and an Immunochip (16–18, 20, 21). This has lead to the discovery of a significant number of consistent genetic risk loci, including the HLA region that, as in other vasculitis, is the main susceptibility locus for BD. Specifically, an association between the classical allele HLA-B^*^51 and this disorder has been consistently identified in different ethnic groups during the last years (102). Moreover, dense genotyping and imputation of this region have evidenced additional independent signals. In this regard, a study published in 2013 reported that the association between HLA-B^*^51 and BD was explained by a SNP located between the HLA-B and MICA genes (103). They also identified three independent signals within the HLA region, located within *PSORS1C1* (psoriasis susceptibility 1 candidate 1), upstream HLA-F-AS1 (HLA-F antisense RNA 1), and within HLA-Cw^*^16:02. In addition, a subsequent Immunochip study also described two signals, HLA-B^*^57 and HLA-A^*^03, that showed an independent effect to that conferred by HLA-B^*^51 (18).

Several loci outside the HLA region have also shown robust associations with this vasculitis. The first GWASs on BD, performed in Japanese and Turkish populations and simultaneously published in 2010, evidenced the role of *IL10* and *IL23R*/*IL12RB2* as genetic risk factors in BD (16, 17). The association with the *IL23R*/*IL12RB2* locus was subsequently confirmed in an Immunochip study performed in BD patients from Spain (18). *IL10* encodes a cytokine that has an anti-inflammatory role by suppressing the expression of pro-inflammatory cytokines, such as IL-6, IL-12, and IL-1, but also promotes B cell responses by enhancing B cell survival, proliferation, and antibody production (104). The *IL23R*/*IL12RB2* locus contains two genes with a crucial role in the inflammatory response. *IL23R* encodes a subunit of the IL-23 receptor, whereas *IL12RB2* encodes an IL-12 receptor chain. As previously indicated, IL-12 and IL-23 participate in the immune responses mediated by Th1 and Th17 cells, respectively (57).

In addition, a follow-up study, in which data from the Turkish GWAS were imputed, identified four new loci contributing to the BD susceptibility, *CCR1*/*CCR3* (C-C motif chemokine receptor 1/3), *STAT4* (signal transducer and activator of transcription 4), *KLRC4* (killer cell lectin like receptor C4), and *ERAP1* (endoplasmic reticulum aminopeptidase 1) (19). All these loci play relevant roles in the immune response. The *CCR1* and *CCR3* genes form a chemokine receptor gene cluster, which also includes *CCR2, CCRL2, CCR5*, and *CCXCR1*, on the chromosomal region 3p21. These genes encode proteins critical for the recruitment of effector immune cells to the site of inflammation (105). The protein encoded by *STAT4* is a member of the STAT family of transcription factors that mediates responses to IL-12, IL-23, and type 1 IFNs, and regulates the differentiation of Th1 and Th17 lymphocytes (106). The signal detected at the *KLRC4* region is located within a haplotype block that contains five natural killer (NK) cell receptor genes (*KLRK1, KLRC1, KLRC2, KLRC3*, and *KLRC4*), some of which act as co-stimulators for CD4+ and CD8+ T cells (107). Finally, *ERAP1* encodes an amino peptidase that is crucial for antigen presentation through HLA class I molecules. Interestingly, *ERAP1* variants conferred risk for BD in HLA-B^*^51 positive individuals preferentially, thus suggesting the existence of an interaction between both proteins (19).

In 2013, a third GWAS performed on BD patients from Korea reported *GIMAP4* (guanosine-5′-triphosphatase (GTPase) IMAP family member 4) as a new susceptibility locus (20). This gene encodes a protein belonging to the GTP-binding superfamily and to the immuno-associated nucleotide (IAN) subfamily of nucleotide-binding proteins that seems to play a role in regulating T cell apoptosis (108). Functional studies performed by the authors revealed that the minor allele of the most associated SNP within this region correlated with lower protein activity, and that CD4+ T cells from BD patients have a diminished *GIMAP4* expression (20).

A genome-wide association between *IL12A*, encoding the p35 subunit of IL-12, and BD has also been described in a GWAS performed on an admixed cohort including Western Europeans, Middle Eastern and Turkish cases with BD (21). This association was confirmed in a subsequent Immunochip study, in which the *JRKL*/*CNTN5* (jerky like/contactin 5) locus was also identified as a new genetic risk factor for BD (18). The protein encoded by *JRKL* has an unknown function, whereas *CNTN5* encodes a member of the immunoglobulin superfamily that mediates cell surface interactions during nervous system development (109).

BD treatment has undergone a significant evolution over the years, thanks to the increased knowledge of the pathogenic mechanisms involved in this disease. Genetic findings have evidenced the prominent role of immune responses mediated by Th1 and Th17 cells in BD, with multiple pro-inflammatory molecules contributing to its pathological landscape. This has led to the study of new biological therapies, most of them targeted against cytokines.

In this line, inhibition of IL-1 and IL-6 has shown the most interesting results in both small case series and clinical trials. Specifically, three IL-1 blockers have shown clinical efficacy in BD patients, the IL-1 receptor antagonist anakinra, as well as canakinumab and gevokizumab, targeting the IL-1 molecule directly, which have proved to be effective for all BD manifestations, especially for the most severe ocular involvement (110). In relation to IL-6 inhibition, tocilizumab has proved to be highly effective in treating BD patients with neurological involvement as well as in controlling uveitis, although less promising results were found regarding the treatment of mucocutaneous manifestations (110). Ustekinumab and secukinumab have also shown clinical efficacy in case series studies (111, 112). In addition, the clinical utility of Ustekinumab is currently being evaluated in a phase 2 clinical trial (NCT02648581).

Although there are more clear evidences of T cell involvement in BD, several studies have suggested a possible pathogenic role of B lymphocytes. Indeed, depletion of B cells using rituximab has also emerged as a promising therapy in BD patients (110).

### Kawasaki's Disease

Kawasaki's disease (KD) is a systemic vasculitis that affects small and medium size vessels. It mainly affects children younger than 5 years of age, especially of Asian origin. The most serious complication of KD is the development of coronary artery lesions (CALs), representing the main cause of acquired heart disease among children in Japan, Europe and the USA (113).

Although seven GWASs and an Immunochip have been published in both European and Asian KD cohorts in recent years (22, 24–26, 39, 114–116), only a few consistent genetic associations have been described so far, probably due to the lack of statistical power of most of these studies.

As in other vasculitis, the HLA locus seems to be involved in the KD genetic predisposition. However, contradictory results have been found regarding the specific HLA alleles associated with this disease. Whereas early candidate gene studies identified associations with HLA-Bw54 (previously known as Bw22) in Japanese population (117, 118) and with HLA-Bw51 and HLA-B44 in European patients (119–121), a genetic variant located within the HLA class II region (between HLA-DQB2 and HLA-DOB) was identified as the strongest signal in a GWAS performed on KD cases from Japan (26). This association within the HLA class II region was then replicated in an European-American case-parent trio study using the Immunochip platform (39). In addition, a more recent GWAS identified several SNPs within the HLA class I region associated with KD in Korean population, but failed to replicate this association in an independent case/control set from Japan (24). The small sample sizes of these studies and the varying LD patterns observed across different populations are likely explanations of these heterogeneous findings.

Regarding genetic risk factors outside the HLA locus, genome-wide associations within the *ITPKC* and the *FCGR2A* loci were identified in a GWAS performed in European KD patients and replicated in independent cohorts of Asian and European descent (22). The KD-associated SNP within *FCGR2A* is a functional variant encoding a H131R substitution. It has been reported that the presence of arginine instead of histidine at this amino acid position reduces the affinity of the receptor for the IgG2 isotype (122). *ITPKC* (inositol-trisphosphate 3-kinase C), encoding one of the three isoenzymes of ITPK that phosphorylate inositol 1,4,5-trisphosphate (IP3), was initially implicated in KD by linkage analysis using sib-pairs (123). This same study showed for the first time that ITPKC acts as a negative regulator of T cell activation through the Ca2+/nuclear factor of activated T cells (NFAT) signaling pathway. Interestingly, a subsequent study showed that the genetic variant associated with KD has functional consequences, influencing the ITPKC protein levels, which regulates the production of IL-1β and IL-18 (124). Moreover, using a positional candidate gene study for the 4q35 region, previously linked to KD, Onouchi et al. identified several genome-wide associations within the *CASP3* (caspase 3) gene, which encodes a caspase with a crucial role in apoptosis (23). Similarly to the function identified for *ITPKC*, this study also reported that one of the associated SNPs, located within the 5′ untranslated region of the gene, had functional implications, affecting binding of NFATc2 to the DNA sequence surrounding this polymorphism.

In 2012, two subsequent GWASs, published simultaneously, identified two new susceptibility loci for KD, *BLK* (BLK proto-oncogene, Src family tyrosine kinase) and *CD40* (CD40 molecule) (25, 26). *BLK* encodes a non-receptor tyrosine-kinase of the src family of proto-oncogenes with a crucial role in B cell receptor signaling, thus participating in B-cell activation and antibody secretion (125). The *CD40* gene is a member of the TNF-receptor superfamily that encodes a receptor expressed on antigen-presenting cells involved in inflammation through selection of autoreactive T cells and activation of B and T cells (126).

Additionally, large-scale genetic studies have reported suggestive signals in different loci, including *CAMK2D* (calcium/calmodulin-dependent protein kinase II delta), *CSMD1* (CUB and Sushi multiple domains 1), *LNX1* (ligand of numb-protein X1), *NAALADL2* (N-acetylated alpha-linked acidic dipeptidase-like 2), *TCP1* (t-complex 1), *PELI1* (pellino E3 ubiquitin protein ligase 1), *DAB1* (Dab reelin signal transducer homolog 1), *COPB2* (coatomer protein complex beta-2 subunit), *ERAP1, NMNAT2* (nicotinamide nucleotide adenylyltransferase 2), *FUT1* [fucosyltransferase 1 (H blood group)], *RASIP1* (Ras interacting protein 1), and *BRD7P2* (bromodomain containing 7 pseudogene 2) (24, 39, 114–116). However, these associations did not reach genome-wide significance level nor were replicated in later studies and, therefore, they cannot be considered as established susceptibility loci.

As shown by genetic studies, both T and B cells participate in the pathogenic mechanisms implicated in KD. The involvement of the *FCGR2A* gene evidences the relevant role of IgG receptors in the pathogenesis of this vasculitis, providing a biological basis for the use of intravenous immunoglobulin (IVIG), the standard treatment for KD patients. However, approximately up to 20% of patients do not fully respond to this therapy, presenting an increased risk for the development of coronary aneurysms (127). Therefore, several therapeutic options are being tested for treatment of refractory cases.

Considering the role of TNF-α in the pathogenesis of KD, the clinical efficacy of TNF-α inhibitors, such as infliximab and etanercept, has been evaluated. Although treatment with infliximab has shown clinical efficacy in different studies, including reduction of fever duration, markers of inflammation and immunoglobulin reaction rates, its role on the prevention of CALs is still to be determined (128). A phase III trial comparing the efficacy of a second dose of IVIG with infliximab treatment is currently recruiting participants (NCT03065244). In addition, a phase II clinical trial to determine the safety and efficacy of etanercept in reducing the incidence of persistent or recurrent fever in KD patients is currently ongoing (NCT00841789).

On the other hand, studies in mice have demonstrated that both IL-1α and IL-1β are involved in the development of CALs in KD (129, 130). Interestingly, *PELI1*, encoding a protein that acts an intermediate component in the signaling cascade initiated by the IL-1 receptor (131), showed a suggestive association with CALs development in a KD GWAS (115). Thus, genetic findings also support the role of this pathway as a potential drug target in this vasculitis. Considering this, the potential clinical efficacy of blocking IL-1β receptor using anakinra is currently being explored. Three case reports have reported the beneficial clinical use of this biological agent (128). Moreover, two phase II clinical trials exploring the efficacy of anakinra are currently underway (NCT02179853 and NCT02390596).

### Polyarteritis Nodosa

Polyarteritis nodosa (PAN) is a systemic, necrotizing medium-sized vessel vasculitis, mainly affecting adults between the ages of 40–60 years, although it can also appear in children. The clinical features of PAN depend on the affected organs and include systemic symptoms and involvement of the gastrointestinal, renal and peripheral nervous systems.

In 2014, two independent studies identified loss-of-function mutations in the *CECR1* (cat eye syndrome chromosome region candidate 1) gene, which encodes the extracellular adenosine deaminase 2 (ADA2), using whole-exome sequencing (132, 133). Interestingly, in many cases, both the clinical manifestations and the histological findings of the deficiency of ADA2 (DADA2) were consistent with the diagnosis of PAN, which suggests that DADA2 contributes to the clinical phenotype of this vasculitis. The ADA2 protein is mainly expressed by myeloid cells and plays a role in the proliferation and differentiation of macrophages. In this regard, its deficiency has been linked to an imbalance in monocytes differentiation toward proinflammatory M1 macrophages (133, 134).

Clinical management of patients with DADA2 is challenging. None of the commonly used immunosuppressive drugs have resulted particularly effective. Anti-TNF agents have shown promise in the management of the inflammatory syndrome and vasculitis; however, this therapy is not able to completely control the disease manifestations in all treated patients. Considering that bone marrow–derived monocytes and macrophages are the main source of secreted ADA2, it was hypothesized that hematopoietic stem cell transplantation (HSCT) could be an effective treatment for this condition. In this regard, two studies have reported that HSCT was able to normalize the plasmatic levels of ADA2 and to control the disease manifestations (135–137), thus suggesting that this therapy could represent a definitive treatment of DADA2. In addition, enzyme-replacement therapies have also been considered as a potential treatment for these conditions. However, the results obtained using this strategy have not been entirely satisfactory (138).

## Genetics Determinants of Treatment Response in Vasculitis

The genetic basis of treatment response has only been evaluated in three types of vasculitis so far, KD, AAV, and BD, mainly by means of candidate-gene association studies. This has led to the identification of several potential genetic predictors of treatment efficacy.

Most of the pharmacogenetic studies performed in vasculitis have analyzed genetic variants involved in the resistance to IVIG therapy in KD. As it was already mentioned, this treatment is highly effective, but around 10–20% of patients are resistant and have a higher risk for CALs. Therefore, it is essential to elucidate the causes of this resistance in order to predict the responsiveness of patients during the early stages of the disease.

Polymorphisms previously associated with KD have been evaluated in relation to the IVIG response. A study published in 2013 showed that the risk alleles of the *ITPKC* and *CASP3* susceptibility variants (rs28493229 and rs72689236, respectively) were overrepresented in IVIG resistant patients with respect to responding patients (28). These associations were replicated in a subsequent study (29), thus supporting the role of these genes in the response to IVIG treatment. Interestingly, a functional study demonstrated that the poor response observed in patients homozygous for the risk allele of the *ITPKC* locus correlated with increased cellular production of IL-1β and IL-18 (124).

On the other hand, additional studies have evaluated the implication of candidate genes in the clinical efficacy of IVIG based on their functional role. Given that the anti-inflammatory activity of IVIG is partly mediated through FcgR (139), the role of several genes encoding these proteins have been explored. In this regard, polymorphisms within *FCGR2B, FCGR2C*, and *FCGR3B* have been involved in the response to this drug in different studies performed by the same group (30–32). In addition, genetic variants located within genes encoding chemokine receptors and their ligands, including *CCR5* (C-C motif chemokine receptor 5), *CCL3L1* (C-C motif chemokine ligand 3 like 1), and *CCL17* (C-C motif chemokine ligand 17), as well as genes encoding pro-inflammatory cytokines, such as *IL1B* and *IFNG*, have also been implicated in the IVIG treatment resistance (33–36). Moreover, an association between a polymorphism of the *HMGB1* (high mobility group box 1) locus, involved in inflammation and cell differentiation, and the clinical efficacy of this treatment has been recently reported (37). However, all these associations need to be confirmed in independent studies.

The genetic basis of IVIG response in KD has also been explored through comprehensive large-scale genetic analyses (38–40). Both GWAS and Immunochip data have been used to identify genetic variants associated with IVIG resistance by stratifying KD patients according to treatment response. A polymorphism within the *BCL2L11* (BCL2 like 11) gene showed a specific association at the genome-wide significance level with the subgroup of responder patients in an IVIG response-stratified genome-wide association study (38). The protein encoded by this gene, known as Bim, is an important regulator of the negative selection of B lymphocytes in the bone marrow and of T lymphocytes both in the thymus and the periphery (140). In addition, a very recent GWAS performed in Korean KD patients identified the *SAMD9L* (sterile alpha-motif domain-containing 9-like) gene as a susceptibility factor for IVIG resistance (40). This gene encodes a cytoplasmic protein involved in multiple cellular processes, such as cell proliferation and innate immune responses to viral infections (141). Several suggestive signals that could be involved in the response to IVIG therapy were identified using the Immunochip platform, including an intronic SNP of the *STX1B* (syntaxin 1B) gene and a genetic variant located in the intergenic region of *BAZ1A* (bromodomain adjacent to zinc finger domain 1A) and *C14orf19* (39).

A study published in 2017 developed a genetic model to predict IVIG resistance in KD patients (142). In this study, the additive effect of 11 SNPs associated with IVIG response (*p* < 1 × 10^−05^) was used to calculate a GWAS-based weighted genetic risk score (wGRS). A significant association between wGRS and the response was found, suggesting that this scoring system can significantly increase the sensitivity and specificity of prediction of IVIG responsiveness.

Regarding AAV, the advances achieved in its therapeutic management in recent years have allowed these forms of vasculitis to go from presenting high mortality to becoming chronic diseases. Currently, the standard treatment for AAV consists of glucocorticoids together with cyclophosphamide or rituximab. However, despite the success of this therapy, a high percentage of patients do not reach complete remission.

Only three pharmacogenetic studies have evaluated the role of genetic variants as predictors of treatment response in AAV so far. One of them, performed in 152 AAV patients from China, was focused on analyzing the possible implication of the HLA locus in the response to remission induction therapy after 6 months (41). Among the 56 HLA-DRB1, HLA-DPB1, HLA-DQB1, and HLA-DQA1 alleles analyzed, HLA-DRB1^*^0405 appeared to be associated with the clinical efficacy of this treatment; specifically, the proportion of patients showing treatment failure was higher in the subgroup of patients carrying this allele (41.7%) than in the subgroup of patients negative for HLA-DRB1^*^0405 (12.9%).

On the other hand, the main mechanism through which rituximab achieves B cell depletion is antibody-dependent cell mediated cytotoxicity (ADCC), which is mediated through FcgR. Regarding cyclophosphamide, it requires activation by the hepatic cytochrome P450 (CYP) enzymes. Considering this, a more recent study has explored the role of several polymorphisms, located within three genes encoding FcgR (*FCGR2A, FCGR2B, FCGR3A*) and two genes encoding different CYP isoforms (*CYP2B6* and *CYP2C19*), in the response to the treatment with rituximab and cyclophosphamide, respectively (42). When both subgroups of patients (96 treated with rituximab and 93 with cyclophosphamide) were individually analyzed, the authors did not find any potential predictor of treatment response among the genetic variants selected. However, when AAV patients were considered as a global cohort, the *FCGR2A* 519AA genotype was found to predict complete response independently of the induction treatment used.

In addition, a study published in 2017 evaluated the role of several candidate genes in the rituximab response in two independent cohorts of patients with AAV, including MPA and GPA (43). Only one (rs3759467) of the 18 analyzed SNPs showed a consistent association with treatment efficacy. Interestingly, this association was specific for the subgroup of patients PR3-ANCA positive. The associated SNP is located in the 5′ regulatory region of the *TNFSF13B* gene, encoding the B-cell activating factor BAFF, which has been reported to increase the production of PR3-ANCA in GPA patients (99), as previously mentioned.

Finally, pharmacogenetic studies performed in BD were focused on analyzing genetic factors implicated in the response to colchicine. This drug is the most frequently and widely used for oral and genital ulcers, papulopustular lesions, and arthralgias; however, some patients do not respond to this therapy.

Until now, two genes have been associated with colchicine response in BD. A study published in 2012 identified an association between two SNPs, C3435T and G2677T/A, of the *ABCB1* (ATP binding cassette subfamily B member 1) gene and the efficacy of this treatment in a candidate-gene study including a cohort of 68 responder and 37 non-responder patients (44). *ABCB1*, also known as *MDR1* (multidrug resistance), is implicated in drug metabolism by encoding an ATP-dependent drug efflux pump for different xenobiotic compounds, including colchicine (143).

A second pharmacogenetic study, in which 165 responder and 215 non-responder patients were analyzed, reported a role of the *MTHFR* (methylenetetrahydrofolate reductase) locus in the response to colchicine treatment (45). This gene encodes an enzyme that catalyzes the conversion of 5,10-methylenetetrahydrofolate to 5-methyltetrahydrofolate, a co-substrate for homocysteine remethylation to methionine. In this case, the polymorphism associated with the response, C677T, causes an amino acid substitution from alanine to valine leading to reduced activity and increased thermolability of the enzyme, which in turn results in increased levels of homocysteine (144). It has been described the existence of hyperhomocysteinemia in BD patients, which correlates with thrombosis and ocular involvement (145).

## Shared Genetic Component in Vasculitis

Nowadays, it is widely accepted that autoimmune disorders in general and vasculitides in particular share susceptibility genes and molecular pathways influencing their development (146, 147). Indeed, a large number of susceptibility loci described here are common to different vasculitides. The combination of different diseases as a single phenotype in large-scale studies, such as GWAS and Immunochip, has proven to be very useful in the evaluation of this shared genetic component and in the identification of potential drug targets that could be repurposed in related conditions (148–151).

To date, two studies have been conducted combining different forms of vasculitides. In the first one, Carmona et al. (152) combined data from large-vessel vasculitis, namely GCA and TAK, and found a significant genetic correlation within the *IL12B* locus. Considering this, ustekinumab, which has been successfully used to treat refractory TAK, could be of potential clinical use in GCA. Similarly, Ortiz-Fernandez et al. (153) combined data of different vasculitides (GCA, TAK, AAV, IgA vasculitis, and BD) and identified a common signal within the lysine demethylase 4C (*KDM4C*) gene, which encodes a histone demethylase involved in epigenetic mechanisms and that could be of potential use in the treatment of these conditions.

## Precision Medicine in Vasculitis: From Genetic Findings to Clinical Application

The goal of precision medicine is to maximize treatment efficacy by developing more targeted drugs directed against biological pathways with a pathogenic role in the disease, as well as by optimizing the use of existing drugs, through the *a priori* selection of those patients who will benefit from a certain treatment.

As described in this review, it is now clear that genetic studies offer great potential for understanding the molecular mechanisms involved in vasculitis. Thus, insight into disease pathogenesis is progressively leading to new ways for targeted biologic treatment. Moreover, based on the moderate effects provided by the thousands of genome-wide SNPs identified by GWAS, nowadays it is possible to predict each individual susceptibility by means of the polygenic risk score (PRS) analysis, which have been recently performed in other diseases with remarkable results (154). Currently, PRSs are being calculated for different phenotypes separately and, as a potential next step, parallel calculation and disease comparisons of PRS could reflect shared and opposite mechanisms in different vasculitides. However, although these diseases have benefited from the genome-wide era, genetic studies conducted to date still lack enough statistical power to detect variants with moderate effects and, consequently, only a few consistent genetic risk loci have been identified so far. Therefore, further genetic studies in larger cohorts are crucial to obtain information on the missing heritability of these disorders.

Moreover, in recent years, the translation of GWAS/Immunochip findings into biological insights has been challenging, mainly due to the difficulty of identifying causal variants, as well as by the fact that many of the disease-associated SNPs are located in non-coding regions of the genome. Therefore, substantial effort is needed to move from association signals to understanding the functional implication of the genes. In this sense, integration of genomic data with other–omic information, such as epigenomic and transcriptomic data, has become a useful approach to unravel the mechanisms underlying complex diseases. Thus, a better understanding of the interaction between these factors will allow us to obtain a clearer picture of the molecular network involved in the pathogenesis of vasculitis, so that we may turn basic biological knowledge into targets for new therapeutic approaches.

On the other hand, it is likely that a better use of existing drugs will improve the clinical management of vasculitis. In this regard, prediction of those patients that will respond to a specific drug based on their molecular profiles results essential. Although several genetic variants have been described as potential predictor of drug efficacy, mainly in KD but also in AAV and BD, at present, no validated biological biomarker exists to predict treatment response in vasculitis. Again, large-scale genetic studies including well-powered cohorts will be essential to identify genetic profiles that help to classify vasculitis patients and to guide the selection of the most appropriate therapeutic intervention.

It is, therefore, expected that genetic findings in vasculitis continue to open new ways for targeted biologic therapies and improve the use of existing drugs, which will lead to a more personalized application of treatment in the future. However, multiple issues must be overcome before precision medicine can be effectively implemented, which will necessarily require great collaborative efforts among vasculitis expertise research groups.

## Author Contributions

MA-H and AM wrote this review. MG-G and JM critically read and edited the manuscript.

### Conflict of Interest Statement

The authors declare that the research was conducted in the absence of any commercial or financial relationships that could be construed as a potential conflict of interest. The handling editor declared a past co-authorship with one of the authors JM. The reviewer DM declared a past co-authorship with one of the authors with the authors JM and AM to the handling editor.
